# Supplementary material for: Effect of Chitosan Incorporation on the Development of Acrylamide during Maillard Reaction in Fructose–Asparagine Model Solution and the Functional Characteristics of the Resultants
Source: Polymers (Basel). 2022 Apr 12;14(8):1565. doi: 10.3390/polym14081565 (PMC9031937; doi:10.3390/polym14081565)
Supplement: Supplementary file 1 [file polymers-14-01565-s001.zip › polymers-1619496-supplementary.pdf]

Supplementary

Effect of Chitosan Incorporation on the Development of Acrylamide during Maillard Reaction in Fructose–Asparagine Model Solution and the Functional Characteristics of the Resultants

Hong-Ting Victor Lin [1,2], Yen-Shu Ting [1], Nodali Ndraha [1], Hsin-I Hsiao [1] and Wen-Chieh Sung [1,2,*]

[1] Department of Food Science, National Taiwan Ocean University, Keelung 202301, Taiwan; hl358@mail.ntou.edu.tw (H.-T.V.L.); ysesst93020@gmail.com (Y.-S.T.); nodali@email.ntou.edu.tw (N.N.); hi.hsiao@email.ntou.edu.tw (H.-I.H.)
[2] Center of Excellence for the Oceans, National Taiwan Ocean University, Keelung 202301, Taiwan
* Correspondence: sungwill@mail.ntou.edu.tw; Tel.: +886-2-24622192 (ext. 5129)

Figures

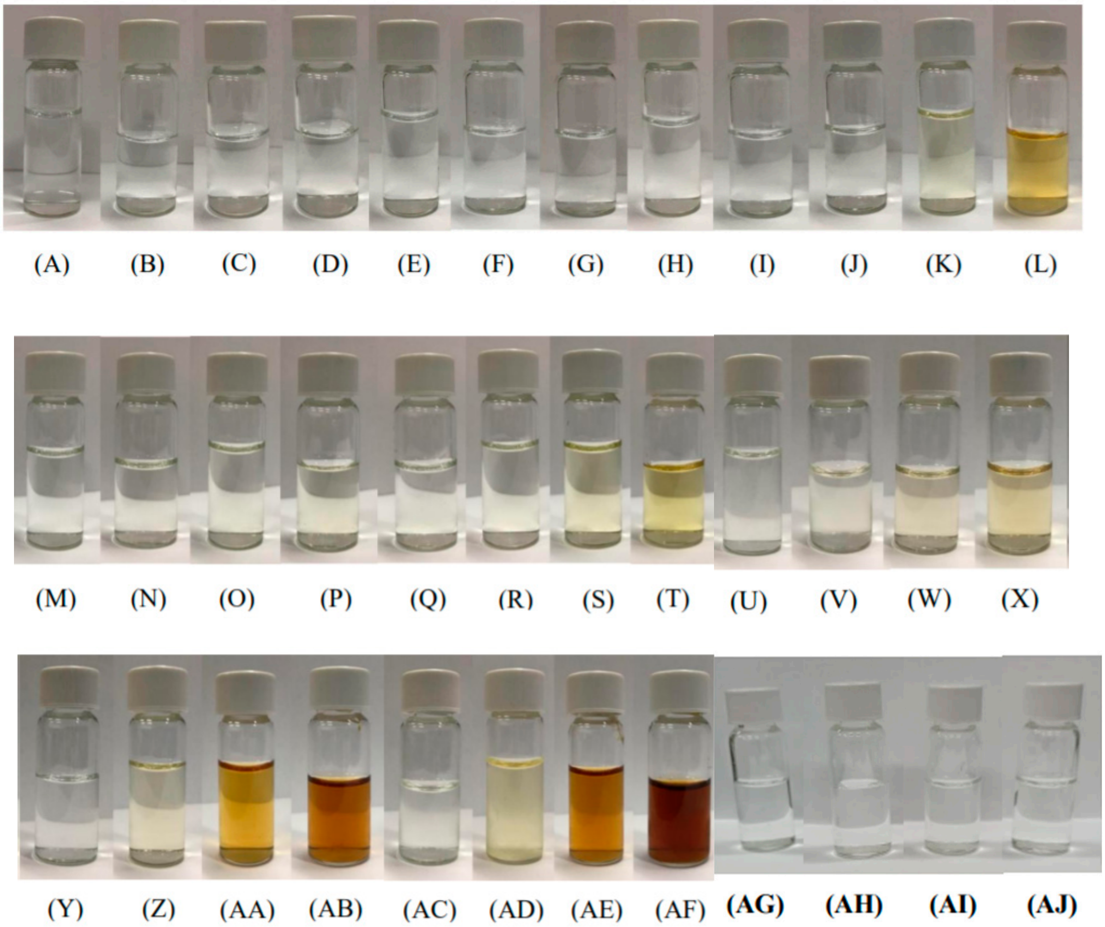

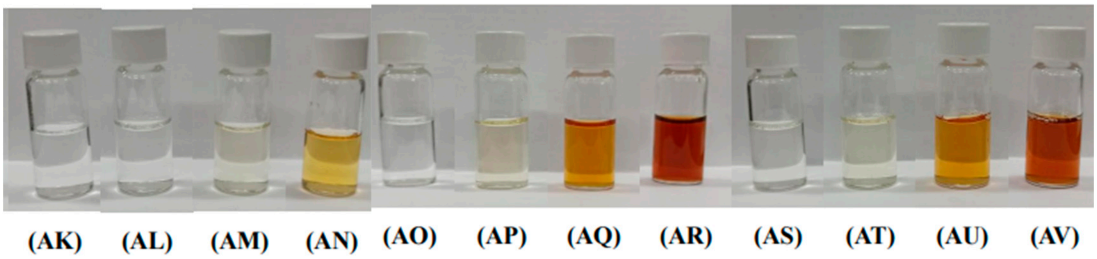

Figure S1. Appearance change during 30 min heating in aqueous solutions containing: glucose: G; asparagine: A; asparagine and glucose: AG; hydroxymethylfurfural: H; hydroxymethylfurfural and asparagine: HA; chitosan: C; asparagine and glucose dissolved in acetic acid: AGV; asparagine, glucose and chitosan dissolved in acetic acid: AGC; asparagine and fructose: AF; fructose: F; asparagine and fructose dissolved in acetic acid: AFV; asparagine, fructose and chitosan dissolved in acetic acid: AFC. (A) G 0 min; (B) G 10 min; (C) G 20 min; (D) G 30 min; (E) A 0 min; (F) A 10 min; (G) A 20 min; (H) A 30 min; (I) AG 0 min; (J) AG 10 min; (K) AG 20 min; (L) AG 30 min; (M) H 0 min; (N) H 10 min; (O) H 20 min; (P) H 30 min; (Q) HA 0 min; (R) HA 10 min; (S) HA 20 min; (T) HA 30 min; (U) C 0 min; (V) C 10 min; (W) C 20 min; (X) C 30 min; (Y) AGV 0 min; (Z) AGV 10 min; (AA) AGV 20 min; (AB) AGV 30 min; (AC) AGC 0 min; (AD) AGC 10 min; (AE) AGC 20 min; (AF) AGC 30 min; (AG) F 0 min; (AH) F 10 min; (AI) F 20 min; (AJ) F 30 min; (AK) AF 0 min; (AL) AF 10 min; (AM) AF 20 min; (AN) AF 30 min; (AO) AFV 0 min; (AP) AFV 10 min; (AQ) AFV 20 min; (AR) AFV 30 min; (AS) AFC 0 min; (AT) AFC 10 min; (AU) AFC 20 min; (AV) AFC 30 min.

Tables

Table S1. The correlation of different heating time between kinematic viscosity、intermediate product、final product、CIELAB color difference and antioxidant activity in the glucose groups.

	Heating time	OD_{294}	OD_{420}	Acrylamide	HMF	Kinematic viscosity	CIE L^*	CIE a^*	CIE b^*	ΔE^*	Fe^{2+} chelating activity	DPPH radical scavenging activity	Reducing power
Heating time	1												
OD_{294}	0.015	1											
OD_{420}	0.426*	-0.125	1										
Acrylamide	0.434*	-0.174	0.971**	1									
HMF	0.001	0.999**	-0.164	-0.213	1								
Kinematic viscosity	-0.235	-0.282	0.002	-0.036	-0.28	1							
CIE L^*	-0.358*	0.089	-0.969**	-0.923**	0.126	-0.002	1						
CIE a^*	0.05	-0.149	0.731**	0.644**	-0.176	0.018	-0.832**	1					
CIE b^*	0.488**	-0.133	0.907**	0.910**	-0.17	-0.005	-0.779**	0.433*	1				
ΔE^*	0.493**	-0.128	0.927**	0.927**	-0.166	-0.011	-0.810**	0.466**	0.999**	1			
Fe^{2+} chelating activity	0.458**	-0.044	0.858**	0.833**	-0.078	0.032	-0.790**	0.435*	0.860**	0.872**	1		
DPPH radical scavenging activity	0.433*	-0.104	0.977**	0.955**	-0.143	-0.027	-0.914**	0.672**	0.937**	0.949**	0.809**	1	
Reducing power	0.410*	-0.169	0.949**	0.943**	-0.208	-0.011	-0.865**	0.572**	0.962**	0.969**	0.864**	0.955**	1

* and ** indicate significance at $p < 0.05$ and 0.01 respectively.

Table S2. The correlation of addition chitosan between kinematic viscosity、intermediate product、final product、CIELAB color difference and antioxidant activity in the glucose groups.

	Chitosan concentration	OD_{294}	OD_{420}	Acrylamide	HMF	Kinematic viscosity	CIE L^*	CIE a^*	CIE b^*	ΔE^*	Fe^{2+} chelating activity	DPPH radical scavenging activity	Reducing power
Chitosan concentration	1												
OD_{294}	0.182	1											
OD_{420}	0.228	0.985**	1										
Acrylamide	0.129	0.983**	0.984**	1									
HMF	0.079	0.960**	0.899**	0.923**	1								
Kinematic viscosity	0.676	-0.289	-0.232	-0.28	-0.388	1							
CIE L^*	-0.298	-0.923**	-0.973**	-0.946**	-0.791*	0.164	1						
CIE a^*	0.289	0.794*	0.884**	0.820*	0.595	-0.064	-0.938**	1					
CIE b^*	0.083	0.939**	0.873**	0.887**	0.974**	-0.322	-0.739*	0.581	1				
ΔE^*	0.107	0.961**	0.905**	0.916**	0.981**	-0.318	-0.784*	0.63	0.997**	1			
Fe^{2+} chelating activity	0.475	0.883**	0.876**	0.818*	0.825*	-0.098	-0.840**	0.731*	0.825*	0.846**	1		
DPPH radical scavenging activity	0.084	0.983**	0.973**	0.983**	0.936**	-0.301	-0.904**	0.801*	0.928**	0.948**	0.796*	1	
Reducing power	0.211	0.980**	0.937**	0.939**	0.986**	-0.316	-0.852**	0.677	0.963**	0.976**	0.900**	0.942**	1

* and ** indicate significance at $p < 0.05$ and 0.01 respectively.

Table S3. The correlation of different heating time between kinematic viscosity、intermediate product、final product、CIELAB color difference and antioxidant activity in the fructose groups.

	Heating time	OD_{294}	OD_{420}	Acrylamide	HMF	Kinematic viscosity	CIE L^*	CIE a^*	CIE b^*	ΔE	Fe^{2+} chelating activity	DPPH radical scavenging activity	Reducing power
Heating time	1												
OD_{294}	0.550**	1											
OD_{420}	0.546**	0.972**	1										
Acrylamide	0.463*	0.894**	0.945**	1									
HMF	0.338	-0.037	-0.076	-0.050	1								
Kinematic viscosity	-0.337	-0.152	-0.131	-0.148	-0.161	1							
CIE L^*	-0.428*	-0.816**	-0.916**	-0.945**	0.041	0.092	1						
CIE a^*	0.032	0.237	0.378	0.469*	0.084	-0.165	-0.668**	1					
CIE b^*	0.529**	0.965**	0.951**	0.877**	-0.105	-0.090	-0.794**	0.140	1				
ΔE	0.526**	0.963**	0.957**	0.888**	-0.110	-0.071	-0.811**	0.152	0.999**	1			
Fe^{2+} chelating activity	0.429*	0.934**	0.884**	0.815**	-0.143	-0.037	-0.716**	0.130	0.939**	0.934**	1		
DPPH radical scavenging activity	0.473*	0.965**	0.916**	0.883**	-0.073	-0.102	-0.752**	0.134	0.960**	0.956**	0.967**	1	
Reducing power	0.619**	0.962**	0.933**	0.861**	0.050	-0.162	-0.747**	0.109	0.939**	0.938**	0.877**	0.937**	1

* and ** indicate significance at $p < 0.05$ and 0.01 respectively.

Table S4. The correlation of addition chitosan between kinematic viscosity、intermediate product、final product、CIELAB color difference and antioxidant activity in the fructose groups.

	Chitosan concentration	OD_{294}	OD_{420}	Acrylamide	HMF	Kinematic viscosity	CIE L^*	CIE a^*	CIE b^*	ΔE	Fe^{2+} chelating activity	DPPH radical scavenging activity	Reducing power
Chitosan concentration	1												
OD_{294}	0.005	1											
OD_{420}	-0.024	0.965**	1										
Acrylamide	-0.267	0.871**	0.936**	1									
HMF	-0.310	0.790*	0.885**	0.985**	1								
Kinematic viscosity	0.766*	-0.453	-0.419	-0.452	-0.421	1							
CIE L^*	0.115	-0.809*	-0.933**	-0.942**	-0.945**	0.346	1						
CIE a^*	-0.151	0.439	0.654	0.675	0.720*	-0.250	-0.856**	1					
CIE b^*	-0.010	0.992**	0.944**	0.848**	0.771*	-0.482	-0.775*	0.394	1				
ΔE	-0.002	0.994**	0.958**	0.871**	0.800*	-0.454	-0.805*	0.426	0.998**	1			
Fe^{2+} chelating activity	0.023	0.944**	0.852**	0.739*	0.615	-0.437	-0.624	0.232	0.929**	0.920**	1		
DPPH radical scavenging activity	-0.096	0.963**	0.882**	0.835**	0.744*	-0.479	-0.688	0.254	0.959**	0.955**	0.960**	1	
Reducing power	-0.064	0.991**	0.955**	0.889**	0.811*	-0.480	-0.807*	0.432	0.976**	0.978**	0.939**	0.976**	1

* and ** indicate significance at $p < 0.05$ and 0.01 respectively.
